# Differences in Antipsychotic-Related Adverse Events in Adult, Pediatric, and Geriatric Populations

**DOI:** 10.7759/cureus.1059

**Published:** 2017-02-26

**Authors:** Hersh Sagreiya, Yi-Ren Chen, Narmadan A Kumarasamy, Karthik Ponnusamy, Doris Chen, Amar K Das

**Affiliations:** 1 Radiology, University of Pittsburgh Medical Center; 2 Department of Neurosurgery, Stanford University Medical Center; 3 Radiology, Montefiore Medical Center; 4 Orthopedics, Western University; 5 Internal Medicine, Stanford University Medical Center; 6 Healthcare and Life Sciences, IBM T.J. Watson Research Center

**Keywords:** antipsychotic drugs, adverse events, pediatrics, geriatrics, fda, drug adverse effects, typical antipsychotics, atypical antipsychotics, database, children

## Abstract

In recent years, antipsychotic medications have increasingly been used in pediatric and geriatric populations, despite the fact that many of these drugs were approved based on clinical trials in adult patients only. Preliminary studies have shown that the “off-label” use of these drugs in pediatric and geriatric populations may result in adverse events not found in adults. In this study, we utilized the large-scale U.S. Food and Drug Administration (FDA) Adverse Events Reporting System (AERS) database to look at differences in adverse events from antipsychotics among adult, pediatric, and geriatric populations. We performed a systematic analysis of the FDA AERS database using MySQL by standardizing the database using structured terminologies and ontologies. We compared adverse event profiles of atypical versus typical antipsychotic medications among adult (18-65), pediatric (age < 18), and geriatric (> 65) populations. We found statistically significant differences between the number of adverse events in the pediatric versus adult populations with aripiprazole, clozapine, fluphenazine, haloperidol, olanzapine, quetiapine, risperidone, and thiothixene, and between the geriatric versus adult populations with aripiprazole, chlorpromazine, clozapine, fluphenazine, haloperidol, paliperidone, promazine, risperidone, thiothixene, and ziprasidone (p < 0.05, with adjustment for multiple comparisons). Furthermore, the particular types of adverse events reported also varied significantly between each population for aripiprazole, clozapine, haloperidol, olanzapine, quetiapine, risperidone, and ziprasidone (Chi-square, p < 10^-6^). Diabetes was the most commonly reported side effect in the adult population, compared to behavioral problems in the pediatric population and neurologic symptoms in the geriatric population. We also found discrepancies between the frequencies of reports in AERS and in the literature. Our analysis of the FDA AERS database shows that there are significant differences in both the numbers and types of adverse events among these age groups and between atypical and typical antipsychotics. It is important for clinicians to be mindful of these differences when prescribing antipsychotics, especially when prescribing medications off-label.

## Introduction

While antipsychotic medications were initially approved based on clinical trials in adult populations, they are commonly prescribed “off-label” in pediatric and geriatric populations [1-2]. In addition, they are increasingly being prescribed to children. Between the 1993 - 1998 and 2005 - 2009 time periods, visits including a prescription for antipsychotics per 100 people increased from 0.24 to 1.83 for children, 0.78 to 3.76 for adolescents, and 3.25 to 6.18 for adults; moreover, antipsychotics were included in 31.1% of youth visits to psychiatrists [3]. While antipsychotics are among the most effective drugs for the treatment of schizophrenia, mania, or acute psychotic reactions, these medications are often prescribed to children and adolescents for non-FDA approved indications, such as disruptive behaviors and aggression [4-5]. Similarly, antipsychotics are frequently used in the elderly and are prescribed to more than a quarter of Medicare patients in nursing homes, with common conditions including dementia, delirium, and behavioral disturbances [2]. However, the use of these medications may result in unanticipated adverse events that are specific to the pediatric and geriatric populations [6]. In our study, we sought to elucidate the differences in adverse events between pediatric, adult, and geriatric populations using the FDA’s Adverse Events Reporting System (AERS), a database that has collected information about adverse events since 1998 [7]. AERS is the FDA’s primary tool for post-marketing adverse event surveillance, with over 250,000 adverse event reports annually [8]. A key strength of the AERS database is the ability to analyze a massive dataset and discover potentially new information regarding drug-related adverse events warranting further investigation. For instance, a recent paper probed the AERS database and found a potential link between amisulpride, cyamemazine, and olanzapine and torsadogenic risk [9]. Drug manufacturers are required to submit adverse event reports, while healthcare providers can voluntarily submit information.

## Materials and methods

We initially imported AERS quarterly data from January 2004 to September 2008 into the MySQL program (v.1.2.17) (Oracle Corp., Redwood Shores, CA). A table was created that mapped all the various drug names for antipsychotics to a generic name and a drug class (typical vs. atypical) using RxNorm (U.S. National Library of Medicine, Bethesda, MD) and Micromedex® (Truven Health Analytics, Greenwood Village, CO) (Table 1). Next, we joined this table with the AERS drug table (matching by DRUGNAME), the AERS demo table (matching by ISR, which stands for individual safety report), and the AERS REAC table (also matching by ISR). We first retrieved the total number of adverse events associated with each drug name, generic name, and drug class. Next, we created a yearage variable (which standardized all ages in AERS to be reported in years using the AGE and AGE_COD variables) as well as the GNDR_COD variable (which was reported as either “M” or “F”) in order to repeat this analysis on the following five subgroups: yearage < 18 (pediatrics), 18 ≥ yearage ≤ 65 (adults), and yearage > 65 (geriatrics), GNDR_COD = “M” (males), and GNDR_COD = ”F” (females).

**Table 1:** List of Antipsychotic Medications Mapped to Generic Name and Drug Class

Drug Name	Generic Name	Drug Class
Abilify	Aripiprazole	Atypicals
Aripiprazole	Aripiprazole	Atypicals
Chlorpromazine	Chlorpromazine	Typicals
Clozapine	Clozapine	Atypicals
Clozaril	Clozapine	Atypicals
Decazate	Fluphenazine	Typicals
Dozine	Chlorpromazine	Typicals
Fazalco	Clozapine	Atypicals
Fentazin	Perphenazine	Typicals
Fluphenazine	Fluphenazine	Typicals
Fortunan	Haloperidol	Typicals
Geodon	Ziprasidone	Atypicals
Haldol	Haloperidol	Typicals
Haloperidol	Haloperidol	Typicals
Invega	Paliperidone	Atypicals
Kentace	Haloperidol	Typicals
Largactil	Chlorpromazine	Typicals
Loxapac	Loxapine	Typicals
Loxapine	Loxapine	Typicals
Loxitane	Loxapine	Typicals
Mellaril	Thioridazine	Typicals
Mesoridazine	Mesoridazine	Typicals
Moban	Molindone	Typicals
Moditen	Fluphenazine	Typicals
Navane	Thiothixene	Typicals
Noxene	Thiothixene	Typicals
Olanzapine	Olanzapine	Atypicals
Orap	Pimozide	Typicals
Ormazine	Chlorpromazine	Typicals
Permitil	Fluphenazine	Typicals
Perphenazine	Perphenazine	Typicals
Pimozide	Pimozide	Typicals
Primazine	Promazine	Typicals
Prolixin	Fluphenazine	Typicals
Promazine	Promazine	Typicals
Quetiapine	Quetiapine	Atypicals
Rideril	Thioridazine	Typicals
Risperdal	Risperidone	Atypicals
Risperidone	Risperidone	Atypicals
Serenace	Haloperidol	Typicals
Serentil	Mesoridazine	Typicals
Seroquel	Quetiapine	Atypicals
Sparine	Promazine	Typicals
Stelazine	Trifluoperazine	Typicals
Symbyax	Olanzapine	Atypicals
Thioridazine	Thioridazine	Typicals
Thiothixene	Thiothixene	Typicals
Thorazine	Chlorpromazine	Typicals
Trifluoperazine	Trifluoperazine	Typicals
Trilafon	Promazine	Typicals
Vesprin	Triflupromazine	Typicals
Ziprasidone	Ziprasidone	Atypicals
Zyprexa	Olanzapine	Atypicals

Next, for each drug, we computed the percent of antipsychotic-related adverse events that the drug represented in each population. We then used the z-test of proportions to compare this percent for each drug in the following categories: pediatrics vs. adults, adults vs. geriatrics, and males vs. females. This process was conducted separately for typical and atypical drugs. This resulted in a z-score and a p-value for each comparison, which was then adjusted using a Bonferroni correction for multiple comparisons, making the significance threshold 0.05/26 = 1.92 x 10^-3^.

Afterward, we retrieved the count of each individual adverse event associated with each generic drug, ordered by the frequency of occurrence in each population. We made sure not to include irrelevant or vague side effects in our top results, excluding terms such as “DRUG INTERACTION,” “ACCIDENTAL EXPOSURE,” and “ACCIDENTAL DRUG INTAKE BY CHILD.” In order to compare the frequencies of the different adverse events in the adult, pediatric, and geriatric populations, we conducted a Chi-square test. For each drug, we selected the top five adverse events in adults and added a sixth column that contained the sum of all other adverse events. We chose the top five since this minimized the number of cells in the Chi-square calculation that contained an expected value less than 5, which is not ideal for the Chi-square test. Next, we compared the frequency of these particular adverse events in the adult, pediatric, and geriatric populations using a 3 by 6 Chi-square table with 10 degrees of freedom, and we calculated p-values for each of seven major drugs—aripiprazole, clozapine, haloperidol, olanzapine, quetiapine, risperidone, and ziprasidone—using the R statistical program (v2.12.2). We also used the MedRDA (Medical Directory for Regulatory Activities: International Federation of Pharmaceutical Manufacturers and Associations, Geneva, Switzerland) hierarchy to map MedDRA Preferred Terms (the default FDA coding) to high-level terms and determined the frequency of the high-level terms in the three populations.

Next, we took the list of the top five adverse events for the seven drugs in the three populations and used Medical Subject Headings (MeSH terms) to evaluate how many times a particular drug-adverse event combination was indexed in PubMed for the three populations. For instance, for the side-effect “TREMOR” for aripiprazole in the geriatric population, we would have used the following search term: *"aripiprazole"[Substance Name] AND ("Aged"[Mesh]) AND tremor*. We then compared the number of reports in AERS and in the literature. For the drug, population, and adverse event combinations that had fewer than five reports in the literature, we manually examined the results to ensure their validity and highlighted the ones that we confirmed to have less than five reports.

## Results

A summary of the populations we studied is shown in Table 2.

**Table 2:** Demographics: Summary of the Population

Category	Value
Total number of patients	61,380
Mean age ± SD	45.7 ± 20.0
Patients where age < 18	3,578
Patients where age ≥ 18 and age ≤ 65	32,660
Patients where age > 65	7,260
Patients where age is not available	17,882
Male patients	27,783
Female patients	29,780
Gender NA (null, unknown, or not specified)	3,817

The percentage of antipsychotic-related side effects was often significantly different in the pediatric, adult, and geriatric populations for atypical and typical antipsychotics as shown in Tables 3-4.

**Table 3:** Comparison of the Number of Adverse Events in Each Population for Atypical Antipsychotics Results were statistically significant either for pediatrics vs. adults, geriatrics vs. adults, or both. The significance threshold was 0.05/26 = 1.92 x 10^-3^. The p-values that R found to be extremely low are labeled as "0." Items that were statistically significant are in bold.

Generic Name	% of Adverse Events			p-value (vs. Adults)		Statistical Significance
	Pediatrics	Adults	Geriatrics	Pediatrics	Geriatrics	
Aripiprazole	25.9	9.8	4.8	0	0	Both
Clozapine	4.6	17.3	13.9	0	2.7e^-14^	Both
Olanzapine	16.1	26.0	25.6	0	0.23	Pediatrics
Paliperidone	0.6	0.6	0.2	0.43	3.5e^-5^	Geriatrics
Quetiapine	24.4	27.2	26.1	2.6e^-5^	0.019	Pediatrics
Risperidone	23.3	14.4	27.5	0	0	Both
Ziprasidone	5.1	4.6	1.9	0.077	0	Geriatrics
TOTALS	100.0	100.0	100.0			

**Table 4:** Comparison of the Number of Adverse Events in Each Population for Typical Antipsychotics Results were statistically significant either for pediatrics vs. adults, geriatrics vs. adults, or both. The significance threshold was 0.05/26 = 1.92 x 10^-3^. The p-values that R found to be extremely low are labeled as "0." Items that were statistically significant are in bold.

Generic Name	% of Adverse Events			p-value (vs. Adults)		Statistical Significance
	Pediatrics	Adults	Geriatrics	Pediatrics	Geriatrics	
Chlorpromazine	20.3	17.1	12.1	0.066	3.31e^-6^	Geriatrics
Fluphenazine	0.3	5.1	2.4	4.3e^-5^	4.39e^-6^	Both
Haloperidol	64.9	56.3	72.8	0.0011	0	Both
Loxapine	2.8	2.7	1.9	0.46	0.043	--
Mesoridazine	0.0	0.2	0.1	0.20	0.27	--
Molindone	0.9	0.3	0.6	0.017	0.018	--
Perphenazine	0.3	2.7	2.3	0.0038	0.19	--
Pimozide	3.4	1.6	1.3	0.0082	0.17	--
Promazine	0.6	2.8	0.1	0.0095	9.39e^-10^	Geriatrics
Thioridazine	5.5	3.9	3.4	0.070	0.20	--
Thiothixene	0.0	4.5	0.9	4.9e^-5^	1.24e^-10^	Both
Trifluoperazine	0.9	2.9	2.0	0.017	0.031	--
TOTALS	100.0	100.0	100.0			

Eight antipsychotics were associated with a significant difference in the number of adverse events in the pediatric vs. adult populations, including aripiprazole, clozapine, fluphenazine, haloperidol, olanzapine, quetiapine, risperidone, and thiothixene. Ten antipsychotics were associated with a significant difference in the number of adverse events in the adult vs. geriatric populations, including aripiprazole, chlorpromazine, clozapine, fluphenazine, haloperidol, paliperidone, promazine, risperidone, thiothixene, and ziprasidone. When we compared the distributions of adverse events in the adult population to the pediatric and geriatric populations, Chi-square tests revealed that they were significantly different, as the p-values were 4.33e^-32^, 1.68e^-92^, 2.60e^-35^, 6.96e^-106^, 4.50e^-124^, 3.43e^-65^, and 1.35e^-7^, respectively, for aripiprazole, clozapine, haloperidol, olanzapine, quetiapine, risperidone, and ziprasidone. Tables comparing the number of reports in the literature to those in the AERS database for the top five adverse events in seven major antipsychotics revealed some outliers in the three populations, as evidenced by the reports with less than five cases in the literature (Tables 5-6).

**Table 5:** Top Adverse Events in the Pediatric Population The searches that have five or less PubMed articles are in bold.

Generic Name	Event Pediatrics	N Pediatrics
Aripiprazole	WEIGHT INCREASED	102
Aripiprazole	TREMOR	86
Aripiprazole	DYSTONIA	82
Aripiprazole	SOMNOLENCE	63
Aripiprazole	EXTRAPYRAMIDAL DISORDER	62
Aripiprazole	OTHERS	3,087
Clozapine	TACHYCARDIA	37
Clozapine	GRANULOCYTOPENIA	32
Clozapine	SOMNOLENCE	31
Clozapine	WHITE BLOOD CELL COUNT DECREASED	26
Clozapine	SEDATION	21
Clozapine	OTHERS	1,069
Haloperidol	SOMNOLENCE	35
Haloperidol	TREMOR	23
Haloperidol	EXTRAPYRAMIDAL DISORDER	18
Haloperidol	MUSCLE SPASMS	15
Haloperidol	NEUROLEPTIC MALIGNANT SYNDROME	14
Haloperidol	OTHERS	770
Olanzapine	WEIGHT INCREASED	106
Olanzapine	AGGRESSION	69
Olanzapine	SUICIDAL IDEATION	58
Olanzapine	ABNORMAL BEHAVIOUR	46
Olanzapine	COMPLETED SUICIDE	44
Olanzapine	OTHERS	3,755
Quetiapine	WEIGHT INCREASED	121
Quetiapine	SUICIDAL IDEATION	80
Quetiapine	TACHYCARDIA	74
Quetiapine	CONVULSION	72
Quetiapine	AGGRESSION	70
Quetiapine	OTHERS	4,745
Risperidone	AGGRESSION	112
Risperidone	WEIGHT INCREASED	69
Risperidone	CONVULSION	66
Risperidone	SUICIDAL IDEATION	65
Risperidone	ABNORMAL BEHAVIOUR	54
Risperidone	OTHERS	4,015
Ziprasidone	DYSTONIA	26
Ziprasidone	SUICIDAL IDEATION	25
Ziprasidone	DEPRESSION	20
Ziprasidone	SUICIDE ATTEMPT	20
Ziprasidone	WEIGHT INCREASED	19
Ziprasidone	OTHERS	943

**Table 6:** Top Adverse Events in the Geriatric Population The searches that have five or less PubMed articles are in bold.

Generic Name	Event Geriatrics	N Geriatrics
Aripiprazole	TREMOR	27
Aripiprazole	NEUROLEPTIC MALIGNANT SYNDROME	22
Aripiprazole	PARKINSONISM	21
Aripiprazole	DEATH	18
Aripiprazole	GAIT DISTURBANCE	15
Aripiprazole	OTHERS	1,087
Clozapine	DEATH	174
Clozapine	PNEUMONIA	100
Clozapine	PYREXIA	63
Clozapine	SOMNOLENCE	50
Clozapine	FALL	46
Clozapine	OTHERS	3,117
Haloperidol	AGITATION	78
Haloperidol	CONFUSIONAL STATE	75
Haloperidol	FALL	68
Haloperidol	PYREXIA	67
Haloperidol	DELIRIUM	62
Haloperidol	OTHERS	5,196
Olanzapine	FALL	175
Olanzapine	CONFUSIONAL STATE	142
Olanzapine	DIABETES MELLITUS	138
Olanzapine	CEREBROVASCULAR ACCIDENT	107
Olanzapine	PNEUMONIA	100
Olanzapine	OTHERS	9,494
Quetiapine	FALL	155
Quetiapine	DEATH	111
Quetiapine	CONFUSIONAL STATE	107
Quetiapine	AGITATION	103
Quetiapine	PNEUMONIA	91
Quetiapine	OTHERS	7,377
Risperidone	SOMNOLENCE	161
Risperidone	DEATH	159
Risperidone	CONFUSIONAL STATE	152
Risperidone	FALL	135
Risperidone	ASTHENIA	117
Risperidone	OTHERS	8,770
Ziprasidone	MYOCARDIAL INFARCTION	15
Ziprasidone	COMA	15
Ziprasidone	LOSS OF CONSCIOUSNESS	11
Ziprasidone	SEDATION	11
Ziprasidone	AGITATION	11
Ziprasidone	OTHERS	706

Chi-square analysis was performed to compare the actual distribution of adverse events between the different populations for each drug. Seven commonly prescribed antipsychotics are presented in Table 7: aripiprazole, clozapine, haloperidol, olanzapine, quetiapine, risperidone, and ziprasidone.

**Table 7:** Chi-Square Analysis The distribution of antipsychotic-related adverse events was compared between the pediatric, adult, and geriatric populations for seven major antipsychotics. For each major antipsychotic drug, adverse events were ordered by their frequency in the adult population, the top five were selected (and the rest designated as "other"), and their distribution was compared using the Chi-square test. The resultant p-value is in the final column.

Generic Name	Event Adults	N Peds	N Adults	N Geriatrics	p-Value
Aripiprazole	DIABETES MELLITUS	11	288	10	
Aripiprazole	WEIGHT INCREASED	102	235	13	
Aripiprazole	INSOMNIA	11	227	12	
Aripiprazole	TREMOR	86	177	27	
Aripiprazole	ANXIETY	13	158	4	
Aripiprazole	OTHERS	3,259	14,491	1,124	4.3e^-32^
Clozapine	GRANULOCYTOPENIA	32	611	27	
Clozapine	LEUKOPENIA	11	390	20	
Clozapine	PYREXIA	16	376	63	
Clozapine	DEATH	0	342	174	
Clozapine	TACHYCARDIA	37	309	17	
Clozapine	OTHERS	1,120	29,669	3,249	1.7e^-92^
Haloperidol	DIABETES MELLITUS	0	205	8	
Haloperidol	NEUROLEPTIC MALIGNANT SYNDROME	14	196	44	
Haloperidol	SOMNOLENCE	35	148	41	
Haloperidol	PYREXIA	10	147	67	
Haloperidol	HYPERTENSION	2	128	12	
Haloperidol	OTHERS	814	14,528	5,374	2.6e^-35^
Olanzapine	DIABETES MELLITUS	42	2,197	138	
Olanzapine	WEIGHT INCREASED	106	1,464	64	
Olanzapine	HYPERTENSION	21	906	56	
Olanzapine	PANCREATITIS	27	865	43	
Olanzapine	DIABETES MELLITUS NON-INSULIN-DEPENDENT	6	720	21	
Olanzapine	OTHERS	3,876	62,460	9,834	7.0e^-106^
Quetiapine	DIABETES MELLITUS	39	2,066	55	
Quetiapine	PANCREATITIS	16	871	28	
Quetiapine	WEIGHT INCREASED	121	662	18	
Quetiapine	SOMNOLENCE	70	528	79	
Quetiapine	DIZZINESS	41	473	75	
Quetiapine	OTHERS	4,875	48,314	7,689	4.5e^-124^
Risperidone	DIABETES MELLITUS	20	614	34	
Risperidone	WEIGHT INCREASED	69	390	15	
Risperidone	DEPRESSION	46	327	41	
Risperidone	SOMNOLENCE	47	292	161	
Risperidone	NEUROLEPTIC MALIGNANT SYNDROME	19	262	37	
Risperidone	OTHERS	4,180	32,300	9,206	3.4e^-65^
Ziprasidone	DIABETES MELLITUS	4	243	3	
Ziprasidone	WEIGHT INCREASED	19	173	2	
Ziprasidone	ANXIETY	15	116	5	
Ziprasidone	DEPRESSION	20	105	3	
Ziprasidone	INSOMNIA	12	104	6	
Ziprasidone	OTHERS	983	10,316	750	1.3e^-7^

The top five adverse events for less common drugs are listed in Table 8.

**Table 8:** Number and Type of Events in Each Population for Minor Drugs

Generic Name	Event Pediatrics	N	Event Adults	N	Event Geriatrics	N
Chlorpromazine	DRUG EXPOSURE DURING PREGNANCY	12	DIABETES MELLITUS	100	WEIGHT DECREASED	17
Chlorpromazine	SOMNOLENCE	9	VOMITING	59	DIARRHOEA	16
Chlorpromazine	AGGRESSION	7	NEUROLEPTIC MALIGNANT SYNDROME	52	DEHYDRATION	15
Chlorpromazine	DRUG INEFFECTIVE	6	CONVULSION	51	PNEUMONIA	14
Chlorpromazine	WEIGHT INCREASED	6	PYREXIA	50	SEPSIS	13
Fluphenazine	ARRHYTHMIA	1	DIABETES MELLITUS	26	CONFUSIONAL STATE	6
Fluphenazine	MYOCARDITIS	1	HYPERTENSION	22	URINARY TRACT INFECTION	5
Fluphenazine	PYREXIA	1	WEIGHT INCREASED	17	SOMNOLENCE	5
Fluphenazine	NA	0	HYPONATRAEMIA	17	SEDATION	5
Fluphenazine	NA	0	HYPOTENSION	16	TACHYCARDIA	3
Loxapine	PROTHROMBIN LEVEL DECREASED	3	PYREXIA	15	DYSPHAGIA	5
Loxapine	CONGENITAL GENITOURINARY ABNORMALITY	2	SEPSIS	11	CONFUSIONAL STATE	4
Loxapine	CRYPTORCHISM	2	AGITATION	10	DEPRESSED LEVEL OF CONSCIOUSNESS	4
Loxapine	DRUG EXPOSURE DURING PREGNANCY	2	LACTIC ACIDOSIS	10	SOMNOLENCE	3
Loxapine	RENAL CYST	2	BLOOD CREATINE PHOSPHOKINASE INCREASED	9	ANAEMIA	3
Mesoridazine	NA	0	AGGRESSION	4	MALAISE	1
Mesoridazine	NA	0	EXCESSIVE MASTURBATION	3	STOMATITIS	1
Mesoridazine	NA	0	RASH PAPULAR	3	TARDIVE DYSKINESIA	1
Mesoridazine	NA	0	SKIN ULCER	3	NA	0
Mesoridazine	NA	0	RASH	2	NA	0
Molindone	NEUROLEPTIC MALIGNANT SYNDROME	2	PRESCRIBED OVERDOSE	3	HAEMOGLOBIN DECREASED	3
Molindone	MYOSITIS	1	CONVULSION	3	MYELOID LEUKAEMIA	3
Molindone	PYREXIA	1	DIABETES MELLITUS NON-INSULIN-DEPENDENT	2	PLATELET COUNT DECREASED	3
Molindone	VIRAL MYOSITIS	1	ANGER	2	WHITE BLOOD CELL COUNT INCREASED	3
Molindone	RASH	1	ABDOMINAL DISTENSION	2	TARDIVE DYSKINESIA	3
Paliperidone	NEUROLEPTIC MALIGNANT SYNDROME	10	GALACTORRHOEA	30	DYSPNOEA	4
Paliperidone	HEADACHE	9	EXTRAPYRAMIDAL DISORDER	23	DEEP VEIN THROMBOSIS	3
Paliperidone	CONFUSIONAL STATE	8	AKATHISIA	17	TREMOR	3
Paliperidone	PALPITATIONS	8	OEDEMA PERIPHERAL	17	RENAL FAILURE	3
Paliperidone	DYSTONIA	6	DYSTONIA	12	CONFUSIONAL STATE	2
Perphenazine	NA	0	VOMITING	11	DRUG INEFFECTIVE	6
Perphenazine	NA	0	COMPLETED SUICIDE	10	HYPOTENSION	5
Perphenazine	NA	0	DIABETES MELLITUS	10	INSOMNIA	4
Perphenazine	NA	0	DRUG INTERACTION	8	CEREBRAL INFARCTION	4
Perphenazine	NA	0	DRUG INEFFECTIVE	8	AGRANULOCYTOSIS	3
Pimozide	WEIGHT INCREASED	4	CARDIAC ARREST	12	COMA	4
Pimozide	DIARRHOEA	4	SUICIDE ATTEMPT	8	MEDICATION ERROR	4
Pimozide	RECTAL HAEMORRHAGE	4	DRUG INTERACTION	7	TOXIC SKIN ERUPTION	3
Pimozide	SOMNOLENCE	3	OVERDOSE	6	THROMBOCYTOPENIA	3
Pimozide	ANOREXIA	2	ANXIETY	6	FALL	2
Promazine	NEONATAL DIABETES MELLITUS	1	DIABETES MELLITUS	33	DRUG INTERACTION	2
Promazine	PREMATURE BABY	1	PANCREATITIS	15	METHYLMALONIC ACIDURIA	1
Promazine	DEATH	1	MYOCARDIAL INFARCTION	15	MUSCLE RIGIDITY	1
Promazine	DIAPHRAGMATIC HERNIA	1	BLOOD PRESSURE DECREASED	14	CONFUSIONAL STATE	1
Promazine	PULMONARY HYPOPLASIA	1	MYOCARDITIS	14	PLATELET COUNT INCREASED	1
Thioridazine	NAUSEA	8	HEADACHE	32	CONFUSIONAL STATE	9
Thioridazine	ANOREXIA	8	DIZZINESS	29	HYPERGLYCAEMIA	8
Thioridazine	VOMITING	5	DEPRESSION	25	ANXIETY	7
Thioridazine	ACHOLIA	5	ANXIETY	24	BACK PAIN	7
Thioridazine	AGGRESSION	5	WEIGHT DECREASED	22	DEPRESSION	6
Thiothixene	NA	0	DIABETES MELLITUS	63	DYSPNOEA	4
Thiothixene	NA	0	WEIGHT INCREASED	56	CEREBROVASCULAR ACCIDENT	3
Thiothixene	NA	0	HEADACHE	33	NAUSEA	3
Thiothixene	NA	0	PANCREATITIS	33	TARDIVE DYSKINESIA	3
Thiothixene	NA	0	CHEST PAIN	31	ANTICHOLINERGIC SYNDROME	2
Trifluoperazine	SEXUAL OFFENCE	1	DIABETES MELLITUS	32	CEREBROVASCULAR ACCIDENT	6
Trifluoperazine	CONVULSION	1	INSOMNIA	16	CEREBRAL ATROPHY	5
Trifluoperazine	DEPRESSION	1	DEPRESSION	13	TRANSIENT ISCHAEMIC ATTACK	5
Trifluoperazine	INJURY	1	DIABETIC KETOACIDOSIS	12	TREMOR	5
Trifluoperazine	MEDICATION ERROR	1	PANCREATITIS	12	MYOCARDIAL INFARCTION	4

The top five adverse events for the seven major antipsychotics mapped to MedDRA high-level terms are listed in Table 9.

**Table 9:** Number and Type of Events in Each Population for Major Drugs Organized by MedDRA® High-Level Terms NEC: not elsewhere classified

Generic	Event Pediatrics	N	Event Adults	N	Event Geriatrics	N
Aripiprazole	Neurological signs and symptoms NEC	196	Neurological signs and symptoms NEC	577	Neurological signs and symptoms NEC	56
Aripiprazole	Dyskinesias and movement disorders NEC	172	Dyskinesias and movement disorders NEC	445	General signs and symptoms NEC	36
Aripiprazole	Disturbances in consciousness NEC	143	Anxiety symptoms	420	Muscle tone abnormal	33
Aripiprazole	Physical examination procedures	133	General signs and symptoms NEC	408	Dyskinesias and movement disorders NEC	33
Aripiprazole	General signs and symptoms NEC	117	Physical examination procedures	402	Parkinson's disease and parkinsonism	29
Clozapine	Disturbances in consciousness NEC	78	White blood cell analyses	1,222	General signs and symptoms NEC	193
Clozapine	White blood cell analyses	56	Neutropenias	1,137	Death and sudden death	192
Clozapine	Neutropenias	52	Disturbances in consciousness NEC	952	Disturbances in consciousness NEC	145
Clozapine	Rate and rhythm disorders NEC	42	General signs and symptoms NEC	887	Lower respiratory tract and lung infections	121
Clozapine	Neurological signs and symptoms NEC	39	Neurological signs and symptoms NEC	831	Lower respiratory tract infections NEC	117
Haloperidol	Disturbances in consciousness NEC	51	Disturbances in consciousness NEC	471	Neurological signs and symptoms NEC	246
Haloperidol	Medication errors due to accidental exposures	50	Neurological signs and symptoms NEC	425	Disturbances in consciousness NEC	162
Haloperidol	Muscle tone abnormal	39	General signs and symptoms NEC	381	General signs and symptoms NEC	141
Haloperidol	Dyssomnias	35	Breathing abnormalities	278	Ventricular arrhythmias and cardiac arrest	140
Haloperidol	Dyskinesias and movement disorders NEC	33	Liver function analyses	274	Anxiety symptoms	126
Olanzapine	Suicidal and self-injurious behavior	198	Diabetes mellitus (incl subtypes)	2,403	Disturbances in consciousness NEC	374
Olanzapine	Physical examination procedures	150	Physical examination procedures	2,016	Neurological signs and symptoms NEC	355
Olanzapine	Neurological signs and symptoms NEC	134	General signs and symptoms NEC	1,556	General signs and symptoms NEC	303
Olanzapine	Behavior and socialization disturbances	130	Disturbances in consciousness NEC	1,551	Non-site specific injuries NEC	201
Olanzapine	General signs and symptoms NEC	126	Neurological signs and symptoms NEC	1,312	Liver function analyses	182
Quetiapine	Suicidal and self-injurious behavior	235	Diabetes mellitus (incl subtypes)	2,432	Neurological signs and symptoms NEC	331
Quetiapine	Neurological signs and symptoms NEC	205	General signs and symptoms NEC	1,531	Disturbances in consciousness NEC	292
Quetiapine	Physical examination procedures	178	Neurological signs and symptoms NEC	1,514	General signs and symptoms NEC	221
Quetiapine	General signs and symptoms NEC	172	Disturbances in consciousness NEC	1,386	Non-site specific injuries NEC	181
Quetiapine	Disturbances in consciousness NEC	170	Suicidal and self-injurious behavior	1,120	Circulatory collapse and shock	170
Risperidone	Behavior and socialization disturbances	217	General signs and symptoms NEC	966	Disturbances in consciousness NEC	441
Risperidone	Suicidal and self-injurious behavior	202	Disturbances in consciousness NEC	870	Neurological signs and symptoms NEC	351
Risperidone	Neurological signs and symptoms NEC	194	Neurological signs and symptoms NEC	867	Asthenic conditions	245
Risperidone	General signs and symptoms NEC	128	Diabetes mellitus (incl subtypes)	713	General signs and symptoms NEC	223
Risperidone	Dyskinesias and movement disorders NEC	121	Physical examination procedures	622	Death and sudden death	190
Ziprasidone	Suicidal and self-injurious behavior	61	Neurological signs and symptoms NEC	319	Disturbances in consciousness NEC	41
Ziprasidone	General signs and symptoms NEC	53	General signs and symptoms NEC	300	Ventricular arrhythmias and cardiac arrest	41
Ziprasidone	Behavior and socialization disturbances	52	Anxiety symptoms	283	Neurological signs and symptoms NEC	35
Ziprasidone	Neurological signs and symptoms NEC	47	Diabetes mellitus (incl subtypes)	281	Dyskinesias and movement disorders NEC	24
Ziprasidone	Anxiety symptoms	41	Disturbances in consciousness NEC	280	Ischemic coronary artery disorders	23

## Discussion

Overall, it was evident that both the frequencies and types of adverse events found in the adult population do not fit the distribution found in the pediatric or geriatric populations. As has been seen in prior studies, diabetes mellitus was frequently the most commonly reported adverse event in adults [10], but this was not the case for either the pediatric or geriatric populations. One possible explanation for this is that since adults are more likely than children to have impaired fasting glucose in the first place (often due to a longer exposure to certain physiolologic factors, such as obesity and a sedentary lifestyle), they may be more predisposed to developing this complication. On the other hand, “weight increase” was frequently a top-five adverse effect for the major antipsychotic medications in children, consistent with prior meta-analyses [11]. Children were also more likely to exhibit side effects, such as “aggression,” “abnormal behavior,” and “suicidality,” cognitive effects that may be seen more often in the developing brain. In particular, suicide attempts have previously been linked to antipsychotics in children with the AERS database [12]. For the geriatric population, neurological side effects, such as “confusional state” and “somnolence,” figured more prominently. This suggests that the elderly, who are predisposed to neurological problems, may be more severely affected by the neurological sequelae of antipsychotics. In fact, the Clinical Antipsychotic Trials of Intervention Effectiveness–Alzheimer's Disease (CATIE-AD) trial, studying elderly patients with Alzheimer's disease, showed that atypical antipsychotics were associated with worsening cognitive function comparable to an additional year's worth of cognitive decline compared to placebo [13].

Although we also analyzed differences in high-level terms between pediatric, adult, and geriatric populations, we realized that going to the next higher level grouping for MedDRA terms was not particularly illustrative. For instance, how does one distinguish “Neurological signs and symptoms” from “Disturbances in consciousness,” and what exactly constitutes “General signs and symptoms?” These were among the most commonly reported high-level terms.

Our analysis of the literature revealed that there were adverse events that frequently had reports in AERS; yet, these events were not commonly mentioned in the literature. In the adult population, amongst the top five adverse events for the seven major antipsychotics, only pancreatitis in patients taking quetiapine had fewer than five reports in the literature. The analyses for the pediatric and geriatric populations generated comparatively more adverse events that were not commonly found in the literature. The result for quetiapine in the geriatric population is interesting, given reports of its association with pneumonia [14].

The limitations of the FDA AERS database include the lack of information on the number of individuals taking the various antipsychotic medications in each age group, which could have served as a “denominator” in our study. Due to this lack of a denominator, when comparing the total number of adverse events across the pediatric, adult, and geriatric populations, it was difficult to determine whether variations in the relative distribution of adverse events between the three age groups was truly due to differences in the rate of adverse events rather than simply variations in prescription frequency. For instance, this could be related to prescription trends or when the medications were released. Fortunately, the issue of a denominator was not problematic when comparing the particular side effect profile between the three populations for any given drug. Another issue is the fact that the correlation of a particular medication with an adverse event does not necessarily prove causation. For instance, an individual who is prone to a particular adverse event may be more likely to take an antipsychotic. Another potential problem is recall bias, as a physician who knows a patient is taking a given drug may be more likely to report adverse events that are widely known to be associated with that drug. Nevertheless, the sheer volume of the AERS database and its vast scope make it a useful tool for studying drug-related adverse events.

## Conclusions

Overall, we were able to show that there are significant differences in both the numbers and types of adverse events between the pediatric, adult, and geriatric populations. In addition, this study offers a number of drug and adverse event combinations for follow-up analysis. Given the fact that these medications were overwhelmingly tested on the adult population and are commonly prescribed off-label, it is imperative that clinicians remain mindful of these differences when prescribing these medications in populations for whom the drugs were never formally tested.
